# Z-DNA-Binding Protein 1 Is Critical for Controlling Virus Replication and Survival in West Nile Virus Encephalitis

**DOI:** 10.3389/fmicb.2019.02089

**Published:** 2019-09-11

**Authors:** Hussin A. Rothan, Komal Arora, Janhavi P. Natekar, Philip G. Strate, Margo A. Brinton, Mukesh Kumar

**Affiliations:** Department of Biology, College of Arts and Sciences, Georgia State University, Atlanta, GA, United States

**Keywords:** West Nile virus, flavivirus, Zika virus, Z-DNA-binding protein 1, DNA-dependent activator of IFN-regulatory factors, host-pathogen interaction, virus replication

## Abstract

West Nile virus (WNV), a neurotropic flavivirus, is the leading cause of viral encephalitis in the United States. Recently, Zika virus (ZIKV) infections have caused serious neurological diseases and birth defects, specifically Guillain-Barrè syndrome and microcephaly. Z-DNA binding protein 1 (ZBP1) is a cytoplasmic sensor that that has been shown to play a significant role in initiating a robust immune response. We previously reported that WNV and ZIKV infections induce dramatic up-regulation of ZBP1 in mouse brains as well as in infected primary mouse cells. Herein, we show the critical role of ZBP1 in restricting the pathogenesis of WNV and ZIKV infections. Deletion of ZBP1 resulted in significantly higher morbidity and mortality after infection with a pathogenic WNV NY99 strain in mice. No mortality was observed in wild-type (WT) mice infected with the non-pathogenic WNV strain, Eg101. Interestingly, infection of ZBP1^−/−^ mice with WNV Eg101 was lethal resulting in 100% mortality, suggesting that ZBP1 is required for survival after WNV infection. Viremia and brain viral load were significantly higher in ZBP1^−/−^ mice compared to WT mice. In addition, protein levels of interferon (IFN)-α, and inflammatory cytokines and chemokines were significantly higher in the serum and brains of infected ZBP1^−/−^ mice compared to the WT mice. Primary mouse cortical neurons and mouse embryonic fibroblasts (MEFs) derived from ZBP1^−/−^ mice produced higher virus titers compared to WT cells after infection with WNV NY99 and WNV Eg101. Similarly, neurons and MEFs lacking ZBP1 exhibited significantly enhanced replication of PRVABC59 (Asian) and MR766 (African) ZIKV compared to WT cells. The knockout of ZBP1 function in MEFs inhibited ZBP1-dependent virus-induced cell death. In conclusion, these data reveal that ZBP1 restricts WNV and ZIKV production in mouse cells and is required for survival of a peripheral WNV infection in mice.

## Introduction

Members of the genus *flavivirus* are the most important arthropod-borne viruses causing disease in humans. West Nile virus (WNV) is a neurotropic flavivirus that infects humans, birds, and horses resulting in complex neurological sequelae (Brinton, 2013). WNV infection in humans is usually asymptomatic, but can cause severe neurological disease including meningitis, encephalitis, paralysis, and death (Brinton, 2013; Donadieu et al., 2013). Zika virus (ZIKV) infection can cause fever, headache, fatigue, and neurological symptoms. ZIKV infection is also associated with microcephaly in newborns and Guillain-Barré syndrome in adults (Coyne and Lazear, 2016; Costa and Ko, 2018; Hygino da Cruz et al., 2018; Mehta et al., 2018; Rothan et al., 2018a,b). No anti-viral drugs currently exist for treating patients infected with WNV or ZIKV infection.

Z-DNA binding protein 1 (ZBP1), also called DAI, is one of the cytoplasmic DNA sensors that has been shown to play a significant role in initiating a robust immune response (Schwartz et al., 2001; Ha et al., 2006; Takaoka et al., 2007; Wang et al., 2008). Recent reports demonstrate that ZBP1 senses accumulation of RNA rather than DNA to initiate receptor-interacting protein homotypic interaction motif (RHIM)-dependent activation of receptor-interacting kinase-3 (RIPK3)-dependent necroptosis during HSV-1, murine cytomegalovirus virus (MCMV), influenza virus, and vaccinia virus infections (Kim et al., 2003; Upton et al., 2012; Pham et al., 2013; Wang et al., 2014; Kuriakose et al., 2016; Thapa et al., 2016; Kesavardhana et al., 2017; Koehler et al., 2017). Necroptosis is a form of cell death triggered by RIPK3 phosphorylation that activates the pseudo-kinase MLKL, which upon oligomerization ruptures the plasma membrane, leading to cell death (Wallach et al., 2016). Thus, necroptosis represents a host defense mechanism that combats virus replication in host tissues (Orozco and Oberst, 2017). In addition, recent work has implicated additional roles for ZBP1 and RIPK3 in promoting inflammation, independent of cell death (Daniels et al., 2017, 2019). ZBP1 also regulates NLRP3 inflammasome-mediated production of IL-1β in response to influenza virus infection (Kuriakose et al., 2016). In addition, ZBP1 has been shown to be involved in interferon (IFN) induction in response to HSV-1 (Wang et al., 2008) and human CMV infection (DeFilippis et al., 2010).

We previously reported that WNV and ZIKV infections induce dramatic up-regulation of ZBP1 in mouse brains as well as in infected primary mouse cells (Kumar et al., 2016; Azouz et al., 2019). In the present study, we show the critical role of ZBP1 in restricting the pathogenesis of WNV and ZIKV infections. The ZBP1^−/−^ mice exhibited higher morbidity and mortality after infection with lethal and non-lethal WNV strains compared to wild-type (WT) mice. Primary neuronal cultures and mouse embryonic fibroblasts (MEFs) lacking *Z*BP1 produced higher virus titers after infection with WNV and ZIKV compared to cells derived from WT mice. Collectively, these data provide the first evidence of the requirement for ZBP1 to restrict WNV and ZIKV production and demonstrate that ZBP1-dependent signaling is required to effectively control WNV infection in mice.

## Materials and Methods

### Animals

Wild-type (WT) C57BL/6J mice were purchased from the Jackson Laboratory (Bar Harbor, ME), and ZBP1^−/−^ mice (nbio155) were obtained from the JCRB Laboratory Animal Resource Bank of the National Institutes of Biomedical Innovation, Health and Nutrition (Osaka, Japan). All mice were bred and genotyped in the animal facility at Georgia State University. The WNV infection experiments were conducted in the animal biosafety level-3 laboratory. This study was carried out following the guidelines of the National Institutes of Health and the Institutional Animal Care and Use Committee (IACUC). The protocol was approved by the Georgia State University IACUC (Protocol number A19006).

### Animal Infection Experiments and Plaque Assay

Eight-week-old WT and ZBP1^−/−^ mice were inoculated subcutaneously with 100 plaque-forming units (PFU) of WNV NY99, or 1,000 PFU of WNV Eg101, and the disease symptoms were observed twice daily (Kumar et al., 2013, 2014a,b; Krause et al., 2019). On specific days after inoculation, blood was collected from the tail vein, and serum was separated. In independent experiments, mice were inoculated with PBS (Mock) or WNV NY99 or WNV Eg101 subcutaneously, and on day 8 after inoculation, mice were anesthetized, extensively perfused with PBS, and the brains were harvested. WNV titers in the serum and brain homogenates were measured by plaque assay as described previously (Krause et al., 2019).

### West Nile Virus and Zika Virus Infection of Neuronal Cultures and Mouse Embryonic Fibroblast

Mouse cortical neuron cultures and mouse embryonic fibroblasts (MEFs) were prepared from 1-day-old pups obtained from established colonies of C57/B6J WT and ZBP1^−/−^ mice as described previously (Durkin et al., 2013; Forest et al., 2018; Azouz et al., 2019). The neurons were plated onto poly-D-lysine-coated 6-well or 24-well plates in serum Neurobasal-A medium (Gibco). The cultures were maintained in serum-free Neurobasal A medium supplemented with B27 (Gibco) for 7 days prior to infection. MEFs were grown in DMEM (Gibco) supplemented with 10% heat-inactivated fetal bovine serum and 10 μg/ml gentamicin (Gibco).

Primary neuronal cultures were infected with WNV NY99 at a multiplicity of infection (MOI) of 0.01 and MEFs were infected at a MOI of 1. Both primary neuronal cultures and MEFs were infected with WNV Eg101 at a MOI of 1. For ZIKV infection experiments, neuronal cultures and MEFs were infected at a MOI of 1 with a ZIKV strain, PRVABC59 (Asian strain) or MR766 (prototype African strain). After infection, supernatants and cell lysates were harvested at 24, 48, and 72 h after infection. Virus titers were measured in cell supernatants by plaque assay (Kumar et al., 2013; Kim et al., 2018; Azouz et al., 2019).

### Enzyme-Linked Immunosorbent Assay and Multiplex Immunoassay

Protein levels of IFN-α were measured in the serum and brain homogenates using the VeriKine™ Mouse Interferon-α enzyme-linked immunosorbent assay (ELISA) Kit (PBL Interferon Source) as described previously (Kumar et al., 2012). Multiplex immunoassay kit (MILLIPLEX MAP Mouse Cytokine/Chemokine Kit, Millipore) was used to measure protein levels of inflammatory cytokines and chemokines in the serum (Kumar et al., 2012).

### Quantitative Reverse Transcription-Polymerase Chain Reaction

Virus RNA levels were analyzed in the mouse brains and primary mouse cultures by quantitative reverse transcription-polymerase chain reaction (qRT-PCR). Briefly, total RNA was extracted from homogenized mice brains or cell lysates using a RNeasy Mini Kit (Qiagen) and a iScript™ cDNA Synthesis Kit (Bio-Rad) was used to prepare cDNA samples. Quantitative RT-PCR was used to measure viral RNA levels using primers and probes specific for the WNV or ZIKV as described previously (Kumar et al., 2013, 2017).

### Cell Viability Assay

Neuronal cultures and MEFs seeded in 96-well plates (1 × 10^4^ cells/well) were mock-infected with PBS or infected with WNV NY99. Neuronal cultures were infected at a MOI of 0.01, while MEFs were infected at a MOI of 1. Cell viability was assessed at days 1–3 after infection using a CellTiter 96_AQueous_ One Solution Cell Proliferation Assay (Promega) as described previously (Kumar et al., 2010).

### Statistical Analysis

GraphPad Prism 7.0 was used to perform a Kaplan Meier log-rank test to compare survival curves. Unpaired Student’s *t*-test using GraphPad was used to calculate values of *p* for the clinical scores and plaque assay titers in mouse brains and serum. For plaque assay titers in cell culture supernatants and intracellular viral RNA copies in the cell lysates, two-way analysis of variance (ANOVA) with the *post hoc* Bonferroni test was used to calculate values of *p*. Differences with *p*’s of <0.05 were considered significant.

## Results

### Z-DNA-Binding Protein 1 Signaling Controls West Nile Virus Pathogenesis in Mice Following Peripheral Infection

**Figure 1:**
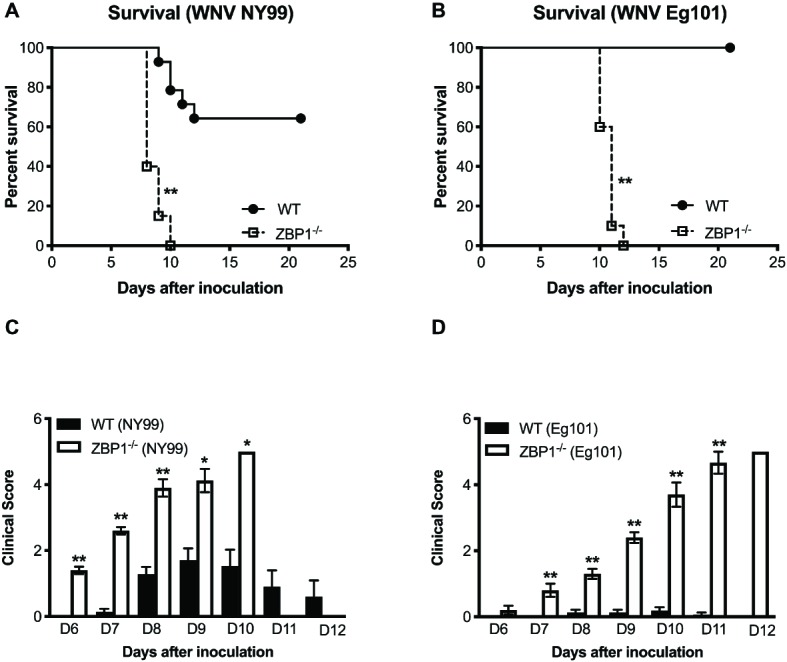
Analysis of survival and clinical score in WT and ZBP1^−/−^ mice following WNV infection. WT and ZBP1^−/−^ mice were inoculated subcutaneously *via* footpad with **(A)** WNV NY99 (100 PFU) or **(B)** WNV Eg101 (1,000 PFU). The difference in the survival of WT and ZBP^−/−^ mice was statistically significant for both WNV NY99 and WNV Eg101 (*n* = 12–22 mice per group). **(C,D)** Animals were monitored twice daily for clinical signs. The designation for the clinical scores is as follows: 1, ruffled fur/hunched back; 2, paresis/difficulty walking; 3, paralysis; 4, moribund/euthanized; and 5, dead. Error bars represent SEM, ^*^p < 0.05, ^**^p < 0.001.

To determine the role of ZBP1 in WNV pathogenesis, we evaluated morbidity of WT and ZBP1^−/−^ mice after WNV infection. Mice were inoculated subcutaneously with either the lethal WNV, strain NY99 (100 PFU) or the non-lethal WNV, strain Eg101 (1,000 PFU). While the infectious dose of 100 PFU of WNV NY99 (Figure 1A) resulted in 40% mortality in WT mice, mortality in ZBP1^−/−^ mice was 100%. The median survival time of infected ZBP1^−/−^ mice was also shorter than in the WT mice. As expected, no mortality was observed in WT mice infected with 1,000 PFU of WNV Eg101 (Figure 1B). Interestingly, infection of ZBP1^−/−^ mice with WNV Eg101 was highly lethal and resulted in 100% mortality. The median survival times observed in WNV Eg101-infected ZBP1^−/−^ mice was similar to the WNV NY99-infected ZBP1^−/−^ mice.

All ZBP1^−/−^ mice developed severe neurological signs after inoculation with WNV NY99 or WNV Eg101 (Figures 1C,D). These clinical signs include ruffled fur, hunchbacked posture, paralysis, tremors, and ataxic gait. WT mice infected with WNV NY99 developed moderate clinical signs while the WT mice infected with Eg101 demonstrated no significant clinical signs. The observation of high morbidity and mortality in ZBP1^−/−^ mice inoculated with WNV Eg101 suggested that ZBP1 is required for survival after WNV infection in mice.

### Z-DNA-Binding Protein 1 Is Required for Control of West Nile Virus Load in the Periphery and Brain

**Figure 2:**
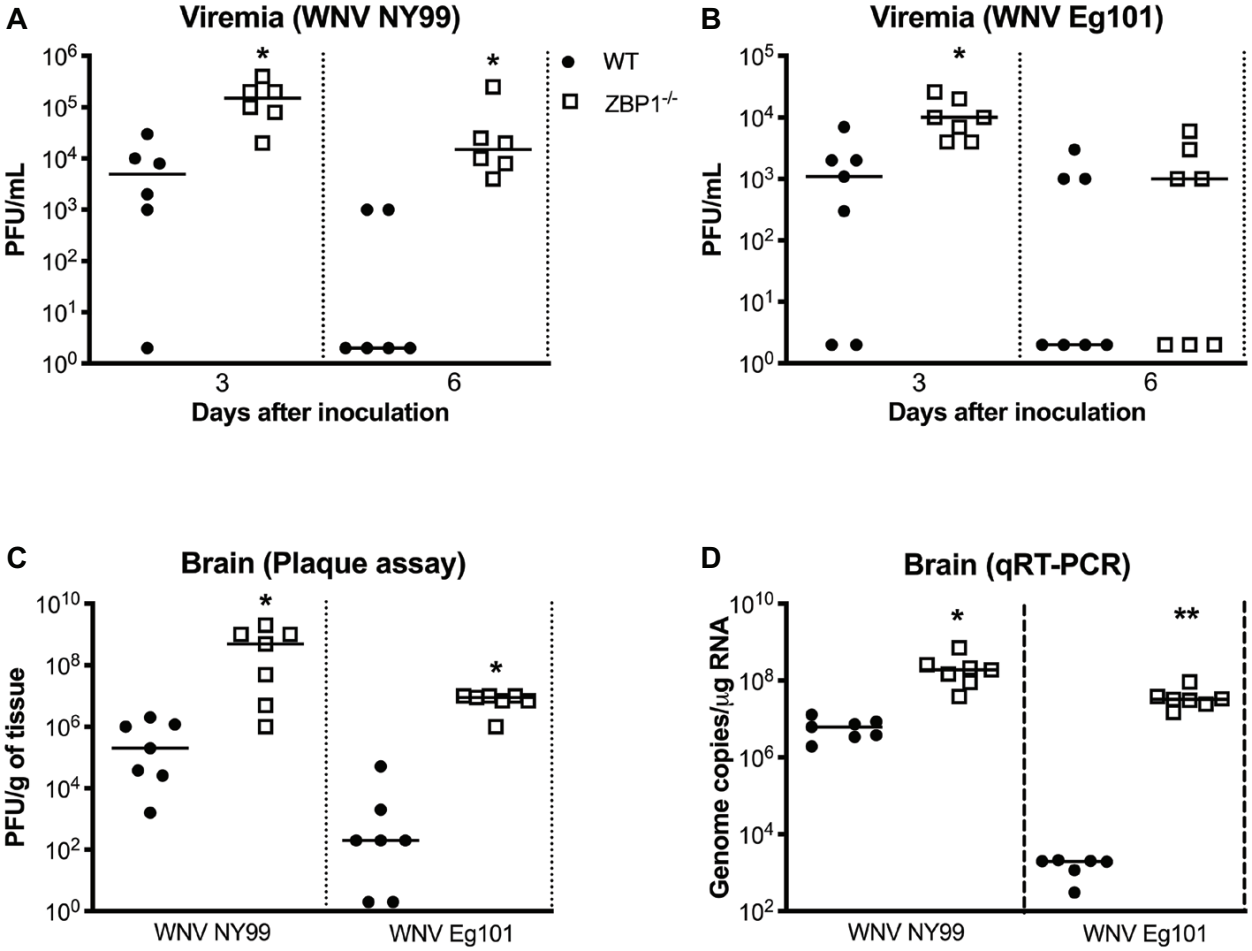
Analysis of virus titers in WT and ZBP1^−/−^ mice. Virus titers were measured in the serum at days 3 and 6 after **(A)** WNV NY99 or **(B)** WNV Eg101 infection by plaque assay and expressed as PFU/mL. **(C)** Virus titers were measured in brain homogenates (day 8 after infection with WNV NY99 or WNV Eg101) and expressed as PFU/g of tissue. **(D)** The WNV RNA copy number in the brain was determined by qRT-PCR and expressed as genome copies/μg of RNA (day 8 after infection with WNV NY99 or WNV Eg101). Each data point represents an individual mouse. The solid horizontal lines signify the median (*n* = 6–7 mice per group). ^*^p < 0.05, ^**^p < 0.001.

We next measured the viral titers in the serum of WT and ZBP1^−/−^ mice at different time-points after subcutaneous WNV NY99 or WNV Eg101 infection. The WNV replication kinetics in the serum of WT and ZBP1^−/−^ mice as measured by plaque assay demonstrated higher viremia in ZBP1^−/−^ mice. WNV titers were significantly higher in ZBP1^−/−^ mice as compared to WT mice at day 3 after WNV NY99 (Figure 2A) or WNV Eg101 infection (Figure 2B). At day 6 post-infection, WNV levels decreased in WT mice, while they remained significantly high in ZBP1^−/−^ mice infected with WNV NY99.

In separate experiments, mice were inoculated with WNV NY99 or WNV Eg101 subcutaneously, and brains were harvested at day 8 after inoculation. It is known that WNV is first detected in the mouse brain around day 6 after subcutaneous inoculation and peak virus load is observed at day 8 after infection. Therefore, we examined viral load in the brains at day 8 after infection. WNV titers in the brain homogenates were measured by plaque assay. WNV load in the brains of ZBP1^−/−^ mice was significantly higher than the WT mice infected with WNV NY99 or WNV Eg101 (Figure 2C). We next measured the WNV RNA copies in the brains of WT and ZBP1^−/−^ mice infected subcutaneously with WNV NY99 or WNV Eg101. WNV RNA copies in the brains of ZBP1^−/−^ mice were significantly higher than the WT mice at day 8 after infection with WNV NY99 (Figure 2D). Very low levels of WNV RNA were detected in the brains of the WT mice infected with WNV Eg101. Nonetheless, significantly higher WNV RNA levels were detected in the brains of ZBP1^−/−^ mice at day 8 after infection (Figure 2D). These data suggest that ZBP1-dependent signaling plays a significant role in controlling WNV load in both the periphery and in the brain.

### Anti-Viral Immune Responses in Wild-Type and ZBP1^−/−^ Mice

**Figure 3:**
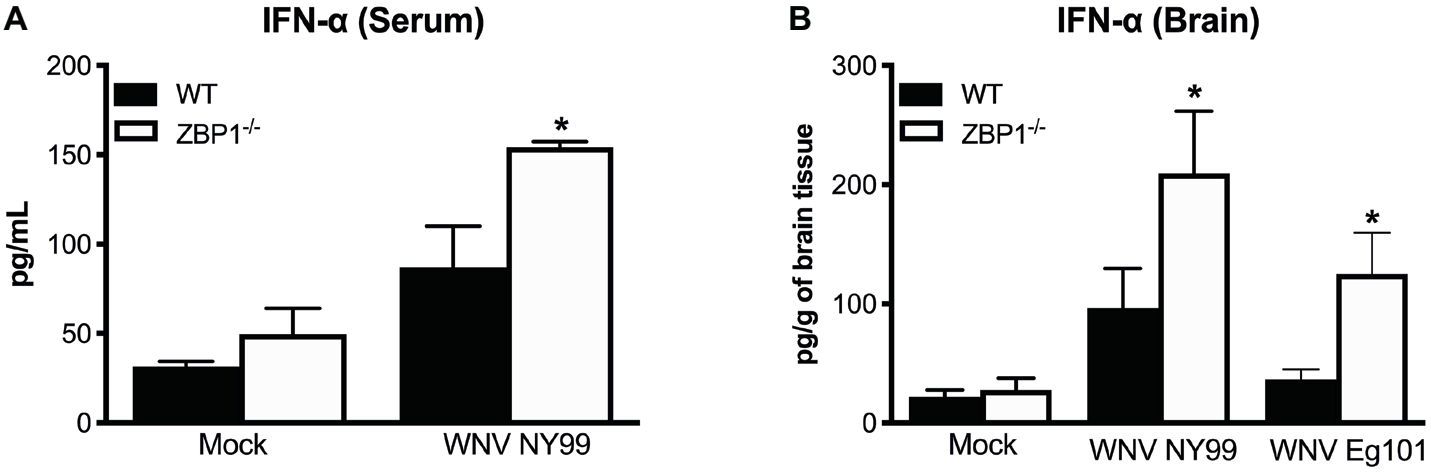
IFN-α levels in the serum and brains of WT and ZBP1^−/−^ mice after infection with WNV NY99 or WNV Eg101. The protein levels of IFN-α were measured in the **(A)** serum and **(B)** brain homogenates using ELISA. Data represent the mean concentration (pg/ml) ± SEM or (pg/g of tissue) ± SEM (*n* = 6–7 mice per group). ^*^p < 0.05.

IFN-α is essential for the WNV clearance from the periphery and in the brain (Suthar et al., 2013). ZBP1 has also been shown to be involved in IFN induction after virus infection (Wang et al., 2008; DeFilippis et al., 2010). Therefore, we measured the protein levels of IFN-α in the serum (day 3 after infection) and brain homogenates (day 8 after infection) of WT and ZBP1^−/−^ mice using ELISA. Our data demonstrate that protein levels of IFN-α in the serum and brain homogenates were significantly higher in ZBP1^−/−^ mice compared to the WT mice (Figure 3).

**Figure 4:**
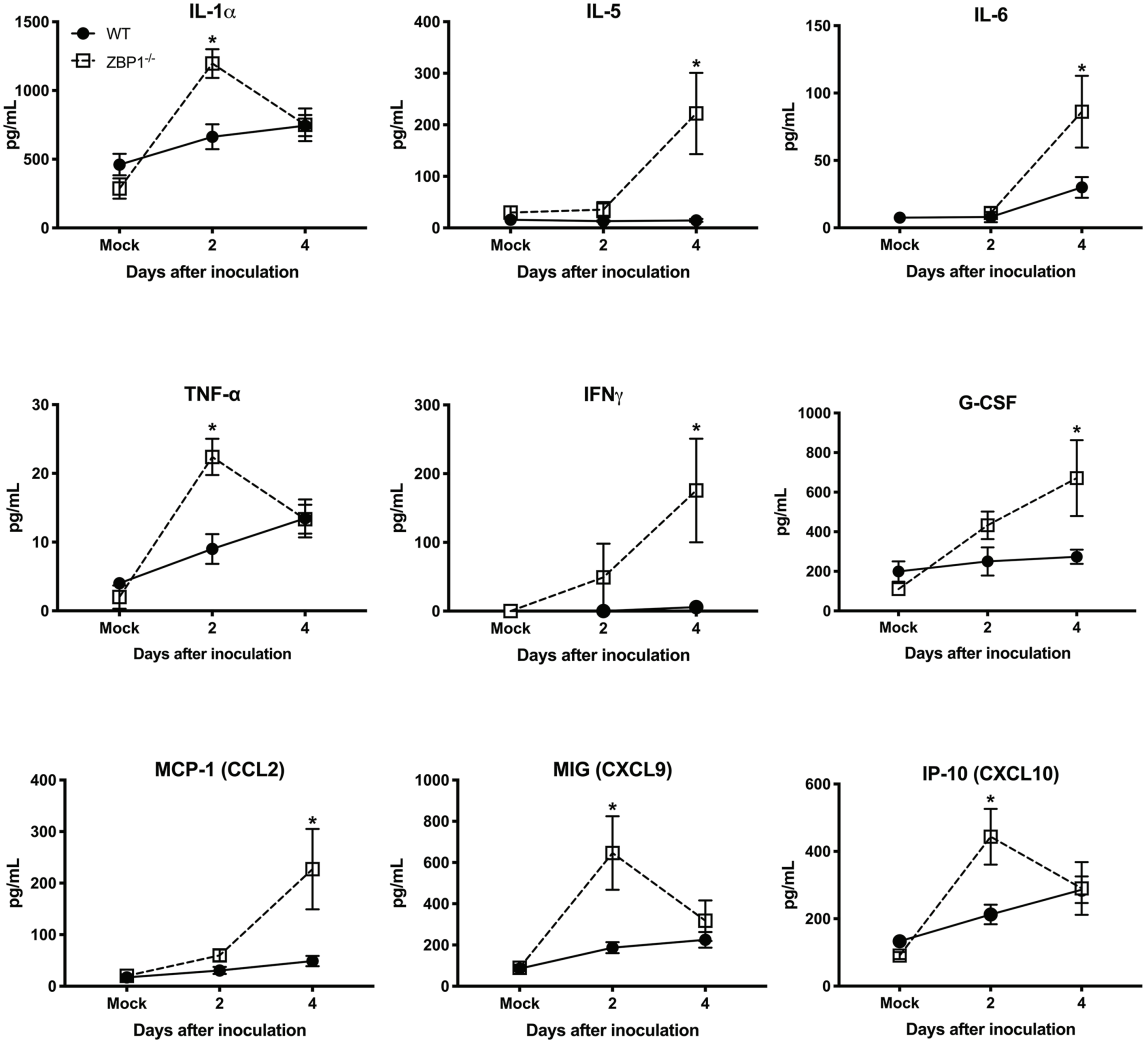
Protein levels of cytokines and chemokines in the serum of WT and ZBP1^−/−^ mice after infection with WNV NY99. Levels of cytokines and chemokines were measured in the serum of WNV NY99-infected WT and ZBP1^−/−^ at days 2 and 4 after infection. Data represent the mean concentration (pg/ml) ± SEM (*n* = 6–7 mice per group). ^*^p < 0.05.

Recent work has implicated a role for ZBP1 in promoting protective inflammation (Daniels et al., 2019). It is known that WNV infection induces a strong up-regulation of multiple cytokines and chemokines. WNV-induced pro-inflammatory mediators are also known to protect mice from lethal WNV disease (Suthar et al., 2013). Therefore, we next assessed the protein levels of key cytokines and chemokines in the serum using a multiplex immunoassay. Protein levels of key anti-viral cytokines and chemokines were significantly higher in the serum of ZBP1^−/−^ mice compared to the WT mice. The protein levels of interleukin (IL)-1α, TNFα, MIG (CXCL9), and IP-10 (CXCL10) were significantly higher in ZBP1^−/−^ mice compared to the WT mice at day 2 after infection (Figure 4). The protein levels of IL-5, IL-6, IFNγ, G-CSF, and MCP-1 (CXCL2) were significantly higher in ZBP1^−/−^ mice compared to the WT mice at day 4 after infection (Figure 4). It is possible that high virus replication in ZBP1^−/−^ mice resulted in a higher inflammatory response. Collectively, these data indicate that ZBP1-mediated restriction of peripheral WNV infection is independent of IFN-α, and anti-viral cytokines and chemokines.

### Z-DNA-Binding Protein 1 Restricts West Nile Virus and Zika Virus Replication in Mouse Cells

**Figure 5:**
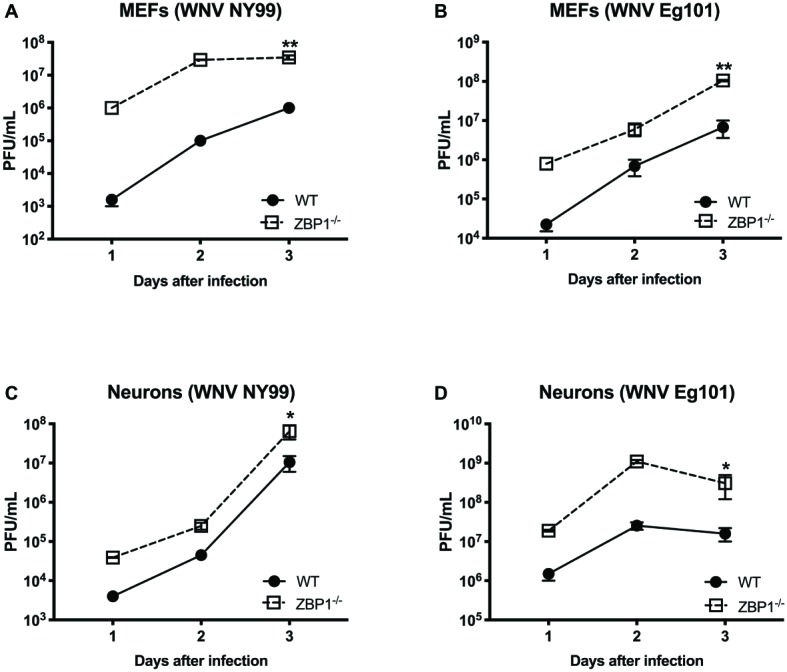
Analysis of virus titers produced by primary cells isolated from WT and ZBP1^−/−^ mice. **(A–D)** MEFs and neuronal cultures were infected with WNV NY99 or WNV Eg101 (as described in Materials and Methods) and virus titers in the cell culture supernatants were measured by plaque assay. Results are expressed as PFU/ml ± SEM from at least three independent experiments conducted in duplicate. ^*^p < 0.05, ^**^p < 0.001.

**Figure 6:**
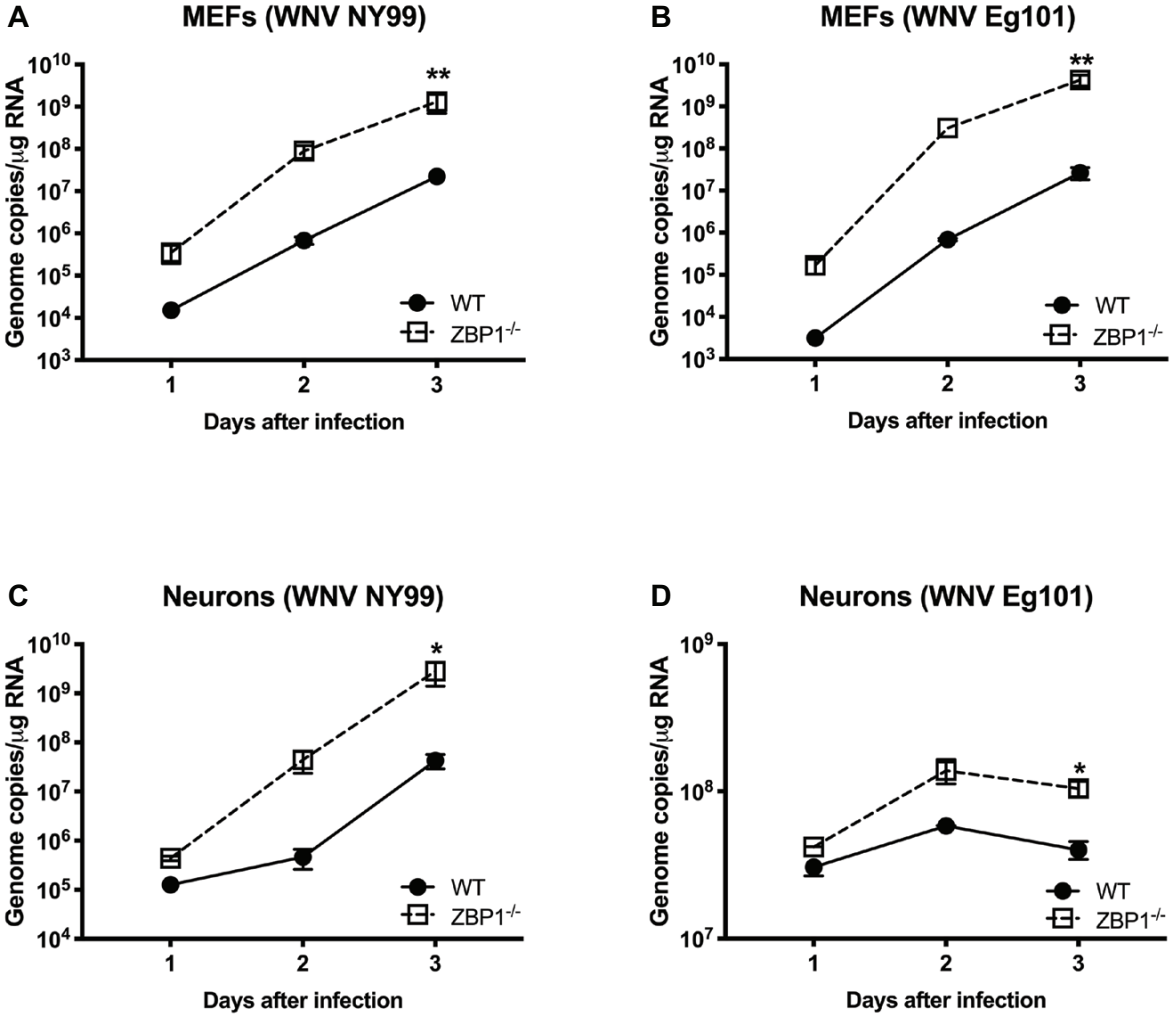
Analysis of viral RNA levels in the primary cells isolated from WT and ZBP1^−/−^ mice. **(A–D)** Cells were infected with WNV NY99 or Eg101 and total RNA extracted from cell lysates was used to conduct qRT-PCR to measure WNV RNA (expressed as genome copies/μg of RNA). Error bars represent SEM (three independent experiments conducted in duplicate), ^*^p < 0.05, ^**^p < 0.001.

To further define the role of ZBP1 during WNV infection, we performed a multistep virus growth analysis in cortical neurons and MEFs isolated from WT and ZBP1^−/−^ mice. Cells were infected with WNV NY99 or WNV Eg101, and supernatants and cell lysates were harvested at 24, 48, and 72 h after infection. Virus titers were measured in cell supernatants by plaque assay. WNV infection of MEFs and neuronal cultures from ZBP1^−/−^ mice resulted in significantly higher virus titers compared to those from WT mice (Figure 5). Total RNA was extracted from the cell lysates and WNV RNA copies were measured using qRT-PCR. Intracellular WNV RNA levels were also significantly higher in cell cultures from ZBP1^−/−^ mice compared to those from WT mice (Figure 6).

**Figure 7:**
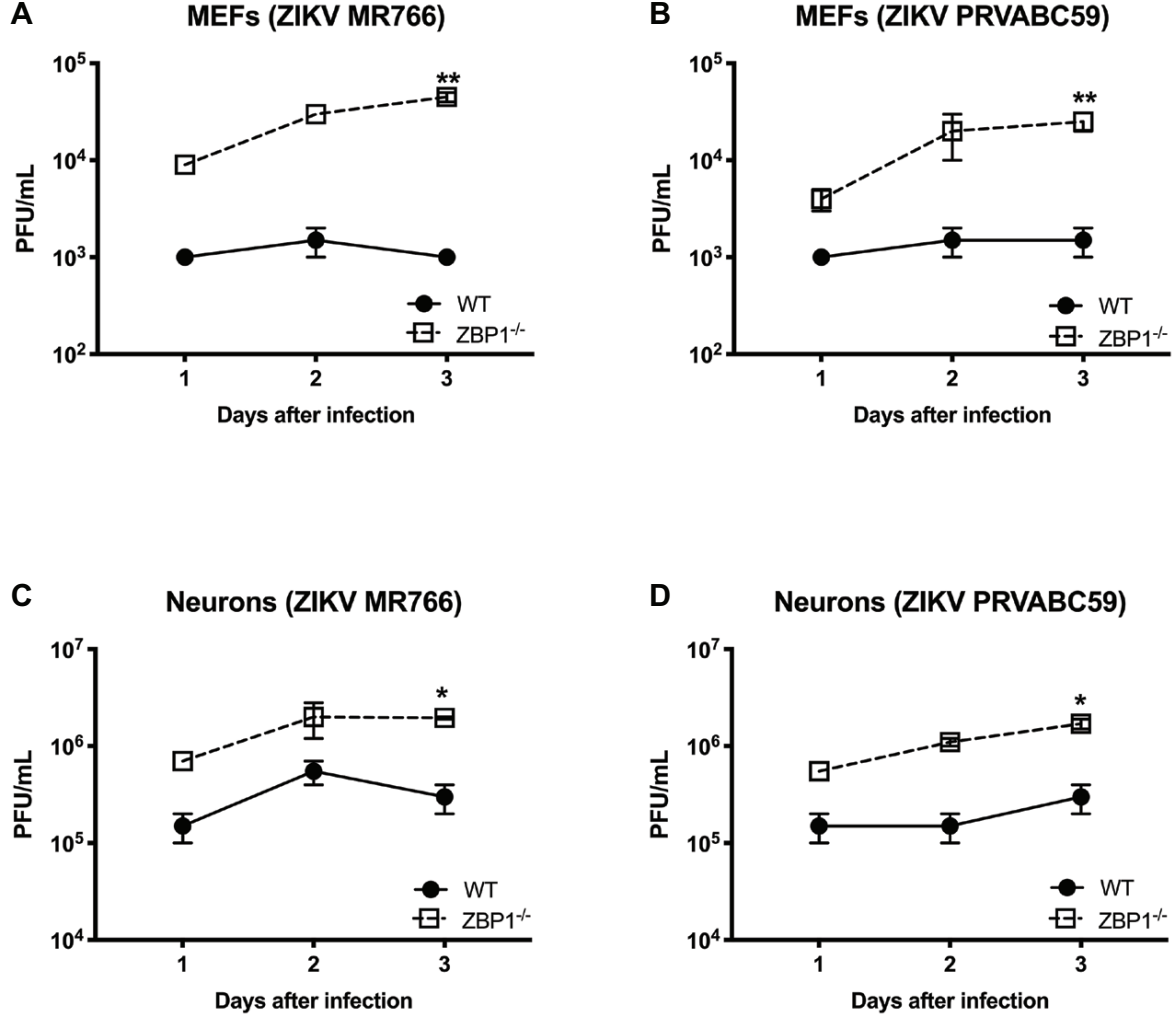
Analysis of ZIKV yields produced by primary mouse cells. **(A–D)** MEFs and neuronal cultures derived from WT and ZBP1^−/−^ were infected with ZIKV MR766 or ZIKV PRVABC59 at a MOI of 1 and virus titers in the cell culture supernatants were measured by plaque assay. Results are expressed as PFU/mL ± SEM from at least three independent experiments conducted in duplicate. ^*^p < 0.05, ^**^p < 0.001.

**Figure 8:**
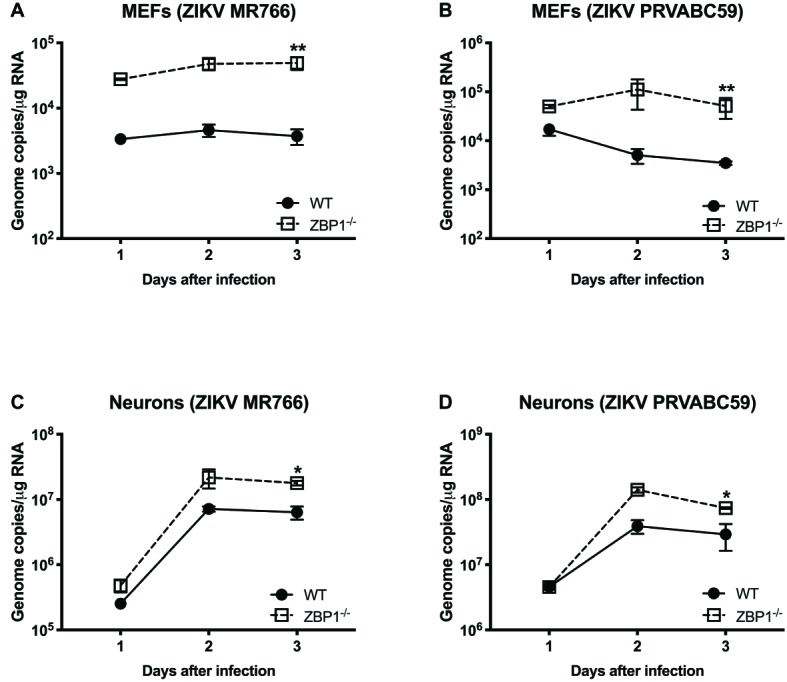
ZIKV RNA levels in the MEFs and neurons isolated from WT and ZBP1^−/−^ mice. **(A–D)** Cells were infected with ZIKV MR766 or ZIKV PRVABC59 at a MOI of 1. Total RNA was extracted from cell lysates and ZIKV RNA was measured by qRT-PCR (expressed as genome copies/μg of RNA). Error bars represent SEM (three independent experiments conducted in duplicate), ^*^p < 0.05, ^**^p < 0.001.

We next examined the role of ZBP1 in ZIKV replication. Similar to WNV, MEFs and neuronal cultures from ZBP1^−/−^ mice produced significantly enhanced virus yields compared to those from WT mice after infection with the Asian or African strains of ZIKV (Figure 7). ZIKV RNA levels were also significantly higher in cell cultures from ZBP1^−/−^ mice compared to those from WT mice (Figure 8). The difference in both WNV and ZIKV titers between WT and ZBP1^−/−^ cells was consistently more dramatic in MEFs (2–3 logs) compared to cortical neurons (1 log). These results correlate with the increased virus titers observed in the serum and brains of ZBP1^−/−^ mice compared to the WT mice after WNV infection.

### Z-DNA-Binding Protein 1-Dependent Cell Death in Primary Mouse Cells Following Infection With West Nile Virus NY99

**Figure 9:**
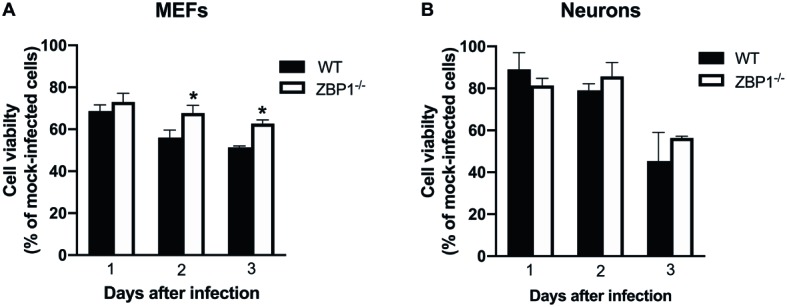
Assay of primary mouse cell viability following WNV infection. **(A)** MEFs and **(B)** neuronal cultures from WT and ZBP1^−/−^ mice were infected with WNV NY99. Cell toxicity on days 1, 2, and 3 after infection was evaluated by cell proliferation assay and the percentage of cell viability was calculated by comparing values to those from mock-infected cells at the corresponding time points. The data are expressed as the mean ± SEM for two independent experiments conducted in triplicate. Error bars represent SEM, ^*^p < 0.05.

MEF and neuronal cultures from WT and ZBP1^−/−^ mice were infected with WNV NY99 and cell viability was measured at 24, 48, and 72 h after infection. Our data demonstrate that the cell viability of infected ZBP1^−/−^ MEFs was significantly higher than that of the WT MEFs at both 48 and 72 h after infection (Figure 9A). However, we did not observe this trend in neuronal cultures as the decrease in WNV-induced cell death was similar in both WT and ZBP1^−/−^ neuronal cultures (Figure 9B).

## Discussion

Our data for the first time demonstrate the critical role of ZBP1 in restricting WNV-induced pathogenesis in mice. ZBP1 reduces WNV and ZIKV production in primary mouse cells and is crucial for survival in the mouse model of WNV disease.

It is known that WNV Eg101 is largely non-pathogenic in adult mice after subcutaneous inoculation (Shirato et al., 2004; Kumar et al., 2014a). However, adult ZBP1^−/−^ mice exhibited 100% mortality after subcutaneous inoculation of WNV Eg101. These data suggest a critical role for ZBP1 in controlling the pathogenic effects of a WNV infection. Viral loads were also significantly higher in the serum and brains of WNV-infected ZBP1^−/−^ mice compared to those of the WT mice. Several previous studies have reported increased virus titers and disease severity in ZBP1^−/−^ mice after infection with influenza virus (Kuriakose et al., 2016; Thapa et al., 2016), HSV-1 (Guo et al., 2018), and MCMV (Upton et al., 2012; Sridharan et al., 2017). One recent study published while this manuscript was in preparation suggests that ZBP1 senses ZIKV infection and restricts disease pathogenesis after intracranial inoculation of ZIKV in mice (Daniels et al., 2019). Another study from the same group previously reported that neuronal RIPK3 signaling is required for survival after subcutaneous WNV infection in mice (Daniels et al., 2017). Our data are in agreement with these observations demonstrating that ZBP1 reduces ZIKV production in primary mouse cells. However, our data for the first time also demonstrate that ZBP1 restricts replication of WNV in mouse cells and is required for survival of a peripheral WNV infection in mice.

Recent work has implicated a role for ZBP1 in promoting protective inflammation. Daniels et al. reported that ZBP1-mediated protective neuroinflammation is required for the protection against intracranial ZIKV infection (Daniels et al., 2019). In the present study, we showed that high virus titers in the serum of ZBP1^−/−^ mice were associated with elevated levels of anti-viral cytokines and chemokines after subcutaneous infection with WNV. Our results are in agreement with previous studies demonstrating high levels of pro-inflammatory cytokines and chemokines in ZBP1^−/−^ mice after infection with *Toxoplasma gondii* and influenza virus (Kuriakose et al., 2016; Pittman et al., 2016). It is known that IFN-α is essential for the WNV clearance from the periphery and in the brain (Suthar et al., 2013). ZBP1 has also been shown to be involved in IFN induction after virus infection (DeFilippis et al., 2010). Our data demonstrate that protein levels of IFN-α in the serum and brain homogenates were significantly higher in ZBP1^−/−^ mice compared to the WT mice. Collectively, these data indicate that ZBP1-mediated restriction of peripheral WNV infection is independent of IFN-α, and anti-viral cytokines and chemokines.

One interesting finding of our study was that we observed a dramatic difference in WNV or ZIKV virus replication in MEFs in the absence of ZBP1. In contrast, deletion of ZBP1 resulted in a modest increase in virus replication in neurons. In addition, we found that the viability of ZBP1^−/−^ MEFs infected with WNV was significantly higher than that of infected WT MEFs. However, the viability of ZBP1^−/−^ neurons was similar to that of WT neurons after WNV infection. These data are in agreement with previous observations demonstrating that ZIKV did not induce ZBP1-dependent cell death in primary neuronal cultures (Daniels et al., 2017, 2019). However, ZBP1-dependent cell death in virus-infected MEFs had never been examined. It is known that activation of the ZBP1-RIPK3 pathway requires high levels of ZBP1 expression and therefore a cell-specific difference in the levels of ZBP1 expression may determine the outcome (Guo et al., 2018). Interestingly, it has been reported that the expression level of ZBP1 is strongly up-regulated in MEFs when stimulated with a synthetic DNA and therefore evokes a stronger innate immune response (Ishii et al., 2006). It could be possible that the ZBP1 and RIPK3 activation is more effective in MEFs compared to neurons. However, more studies are warranted to further understand the cell-specific role of ZBP1 in virus replication.

To our knowledge, our study for the first time revealed a critical role of ZBP1 during peripheral WNV infection in mice. There is need for further mechanistic studies to understand how ZBP1 restricts peripheral WNV and ZIKV infection.

## Data Availability

All datasets generated for this study are included in the manuscript and/or the supplementary files.

## Ethics Statement

The animal study was reviewed and approved by Georgia State University Institutional Animal Care and Use Committee (Protocol number A19006).

## Author Contributions

MK, HR, KA, and MB designed the experiments, analyzed the data, and wrote the manuscript. MK, HR, KA, JN, and PS conducted the experiments. All authors have read and approved the final version of the manuscript.

### Conflict of Interest Statement

The authors declare that the research was conducted in the absence of any commercial or financial relationships that could be construed as a potential conflict of interest.
